# Effect of a Pericardium Graft Inserted Adjacent to the Endplate in the Ahmed Glaucoma Valve Implantation

**DOI:** 10.3390/jcm12196266

**Published:** 2023-09-28

**Authors:** Si Eun Oh, Kyoung In Jung, Hee Jong Shin, Hee Kyung Ryu, Seong Ah Kim, Hae-Young Lopilly Park, Chan Kee Park

**Affiliations:** Department of Ophthalmology, Seoul St. Mary’s Hospital, College of Medicine, The Catholic University of Korea, Seoul 06591, Republic of Korea

**Keywords:** glaucoma drainage device, Ahmed glaucoma valve, pericardium graft

## Abstract

The surface area of encapsulation around the Ahmed glaucoma valve (AGV) endplate is a critical factor in the surgical outcome as it is associated with the degree of IOP reduction. We investigated the surgical outcome of AGV implantation with an additional pericardium graft inserted adjacent to the endplate, with the intent of expanding the surface area of encapsulation. We enrolled 92 patients (92 eyes) who underwent AGV implantation. Of them, 50 patients underwent conventional surgery (termed the without-expansion group), and 42 received an additional an 8 × 6 mm pericardium graft inserted adjacent to the AGV endplate at the sub-Tenon’s space (with-expansion). The hypertensive phase was classified as mild (>21 mmHg), moderate (>25 mmHg), and severe (>30 mmHg). Six months post-surgery, the with-expansion group exhibited a lower IOP (14.90 ± 4.27 mmHg) and lower peak IOP (22.29 ± 4.95 mmHg) than the without-expansion group (17.56 ± 4.88 mmHg and 25.06 ± 6.18 mmHg, *p* = 0.008 and *p* = 0.021, respectively). The with-expansion group exhibited a relatively low rate of moderate (16.7%) and severe (4.8%) hypertensive phases compared to the without-expansion group (40.0% and 20.0%, with *p* = 0.014 and *p* = 0.031, respectively). The additional pericardium graft was associated with a reduced occurrence of moderate hypertensive phase in both univariate and multivariate analysis logistic regression analyses (*p* = 0.017 and *p* = 0.038, respectively). Endplate surface area expansion using an additional pericardium graft reduced the occurrence of moderate and severe hypertensive phases, and lower postoperative 6-month IOP could be achieved.

## 1. Introduction

Glaucoma drainage devices (GDDs) comprise a major intraocular pressure (IOP)-lowering treatment strategy for patients with medically uncontrolled glaucoma [1,2]. GDDs have advantages over trabeculectomy, including less early postoperative complications and postoperative hypotony [3,4]; they are popular even following the introduction of minimally invasive glaucoma surgeries, owing to their IOP-reduction effects. However, the adverse effects associated with GDDs include the development of a hypertensive phase within 3 months of surgery and long-term surgical failure [5,6,7].

Fibrous capsule formation around the endplate of GDDs is a critical factor in the development of the hypertensive phase and affects the main surgical outcome, i.e., IOP reduction; this is because the fibrous capsule around the endplate is the major resistance to the aqueous flow [8]. The fibrous capsule thickness is a factor that influences the success of the Ahmed glaucoma valve (AGV) implantation [9], and the total surface area of encapsulation is another factor associated with the degree of IOP reduction [8,10].

There are several types of GDDs, and the AGV and Baerveldt glaucoma implant (commonly referred to simply as Baerveldt) are two widely used implant types. Both types have advantages in that the AGV is a valved implant with a lower risk of postoperative hypotony, while Baerveldt has a larger endplate, which facilitates greater IOP reduction [7,8]. However, the large endplate of Baerveldt gives rise to complications, such as diplopia, which consequently occurs more often with Baerveldt than with the AGV [11].

We began with the idea of improving the surgical outcome of the AGV while still maintaining safety. We thought of expanding the endplate surface area of an AGV by inserting an additional pericardium graft adjacent to the endplate; a pericardium graft has a soft texture and, hence, is not prone to adverse outcomes such as diplopia. We conducted a longitudinal case–control study of patients who received conventional AGV implantation (without-expansion group) versus patients who received AGV implantation with an additional pericardium graft (8 × 6 mm) adjacent to the AGV endplate (with-expansion group) with a 6-month follow-up. We aimed to compare the surgical outcomes after AGV implantation. We intended to achieve great IOP reduction via surface area expansion of the endplate with the additional pericardium graft while retaining the low risk of hypotony that comes with the valved implant, AGV.

## 2. Materials and Methods

### 2.1. Participants

The present study included 92 patients (92 eyes) who underwent AGV implantation surgery at the Seoul St. Mary’s Hospital between July 2019 and August 2022. Of them, 50 patients underwent the conventional surgery (without-expansion group), while 42 patients received an additional pericardium graft inserted adjacent to the AGV endplate (with-expansion group). 

Our longitudinal case–control study protocol was approved by the Institutional Review Board (IRB) of the Catholic University of Korea (Seoul, Korea) (KC23RISI0103), and the study design followed the tenets of the Declaration of Helsinki. The requirement for written informed consent was waived by the IRB owing to the retrospective nature of this study. 

All participants underwent complete ophthalmic examinations before the surgery, including the slit-lamp examination, Goldmann applanation and noncontact tonometry, measurement of central corneal thickness (UD-800, Tomey Corporation, Nagoya, Japan), red-free fundus photography, retinal nerve fiber layer and ganglion cell/inner plexiform layer thickness using OCT (DRI OCT Triton; Topcon, Tokyo, Japan), and Humphrey visual field examination (Carl Zeiss Meditec, Inc., Dublin, CA, USA). Detailed history taking and review of medical/ocular records were performed to determine the history of systemic diseases, such as hypertension and diabetes mellitus. Lens status, type of glaucoma, and history of previous glaucoma surgery were also recorded. 

Postoperative follow-up examinations were performed on the 1st day; in the 1st and 2nd weeks; and in the 1st, 2nd, 3rd, and 6th months. Slit-lamp examination, IOP measurements using noncontact tonometry, and widefield fundus photography (200Tx, Optos, Dunfermline, UK) were performed at every visit. 

The hypertensive phase was defined as when IOP exceeded 21 mmHg during the first 3 months post-surgery and classified based on the severity into mild (IOP ˃ 21 mmHg), moderate (IOP ˃ 25 mmHg), and severe (IOP ˃ 30 mmHg). 

“Complete success” was defined as achieving a sufficiently low IOP, less than 21 mmHg, without using any IOP-lowering eyedrops. “Qualified success” was defined as achieving an IOP of ˂ 21 mmHg with the use of IOP-lowering eyedrops. “Failure” was defined as IOP over 21 mmHg even with IOP-lowering eyedrops. 

Aqueous suppressants were used as early treatment within 3 months postoperation when postoperative IOP was higher than 18 mmHg to overcome the hypertensive phase by decreasing the fibrosis in the bleb [12].

### 2.2. AGV Implantation

**Figure 1:**
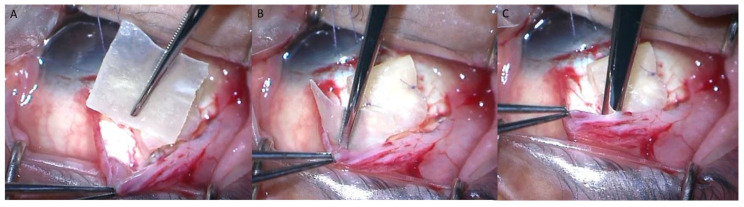
Intraoperative procedures on a right eye; additional pericardium graft inserted adjacent to the Ahmed glaucoma valve (AGV) body, implanted at the superotemporal quadrant. (**A**) The additional pericardium graft sized 8 mm × 6 mm. (**B**,**C**) Pericardium graft was inserted in the sub-Tenon’s space adjacent to the AGV body.

The AGV implantation surgery was performed by two glaucoma specialists (C.K.P. and K.I.J.) using the Ahmed glaucoma valves Model FP7 (New World Medical, Ranch Cucamonga, CA, USA). The AGV implantation was performed using a standard surgical technique. A fornix-based incision was made through the conjunctiva and Tenon’s capsule in the superior temporal quadrant, while radial-relaxing incisions were made on one side of the conjunctival flap to improve surgical exposure. After the valve was primed with a balanced salt solution, the endplate was inserted in the sub-Tenon’s space located 8 mm posterior to the corneoscleral limbus and secured to the sclera with absorbable sutures. A track was made 1–2 mm posterior to the corneoscleral limbus to insert the tube into the anterior chamber with a 1 mm knife. A viscoelastic solution was injected into the anterior chamber using a 23-gauge needle. Subsequently, the tube was cut and inserted into the anterior chamber and secured to the sclera using 8-0 Vicryl sutures (Johnson & Johnson, New Brunswick, NJ, USA). Partial ligation of the tube was performed with a releasable suture using 8-0 Vicryl to reduce the rate of early postoperative hypotony. The extraocular portion of the tube was covered with two layers of 4 × 4 mm pericardium allograft (Maxxeus, Kettering, OH, USA), which was an 8 × 4 mm pericardium patch folded in half. An additional step was performed in the with-expansion group by inserting an 8 × 6 mm pericardium graft adjacent to the AGV body at the sub-Tenon’s space (Figure 1). The conjunctiva was approximated and sutured using 8-0 Vicryl sutures. Topical antibiotics and steroids were administered following the surgery. The releasable suture was removed at 1 week post-surgery when there was no sign of hypotony. The only surgical step that was different between the groups was the insertion of the additional 8 × 6 mm pericardium graft adjacent to the AGV body; all the other steps in the surgery were identical between the two groups. 

### 2.3. Bleb Width

**Figure 2:**
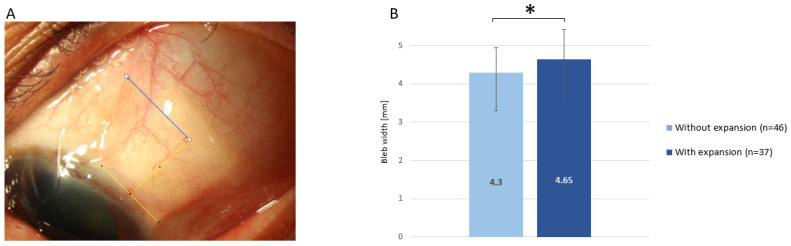
(**A**) Measurement of the bleb width, which was defined as the clearly visible half of the bleb at a 4 mm distance (perpendicular) from the limbus (blue line). (**B**) Bleb width in the without-expansion and with-expansion groups at 3 months postoperation. The bars represent mean ± standard deviation. * *p* < 0.05.

Bleb width was measured at 3 months postoperation by one observer (S.E.O.) in a masked fashion. It was measured as the clearly visible half of the bleb from the center of the bleb where the tube was located, 4 mm perpendicular from the limbus (Figure 2A). The widest bleb width was difficult to measure accurately due to the depth at its location; therefore, this parameter was measured at the 4 mm point, which was considered to mark the beginning of the bleb. The 4 mm width of pericardium covering the tube was used as the reference to measure the 4 mm point perpendicular from the limbus. In most patients, only one side of the bleb margin was visible; however, the wider width was measured when both the margins were visible at the 4 mm point. The bleb width could not be properly measured for nine participants, including four from the without-expansion and five from the with-expansion groups; these patients had either a very small eye or exhibited poor cooperation to facilitate the measurements.

### 2.4. Statistical Analysis 

The Student’s *t*-test and chi-square test were used to compare the variables between the two study groups, and the values were presented as mean ± standard deviation. Univariate and multivariate logistic regression analyses were performed to identify the factors associated with the moderate hypertensive phase, with IOP over 25 mmHg.

## 3. Results

A total of 92 patients (92 eyes) were included, 50 patients in the conventional AVG implantation group (without-expansion group) and 42 in the additional pericardium graft group (with-expansion group) (Table 1). All the baseline clinical characteristics, including age, sex, lens status, and number of preoperative glaucoma medications, were comparable between the two groups, except that more patients in the with-expansion group had undergone prior glaucoma surgeries (57.1% in the with-expansion group vs. 32.0% in the without-expansion group; *p* = 0.04).

**Figure 3:**
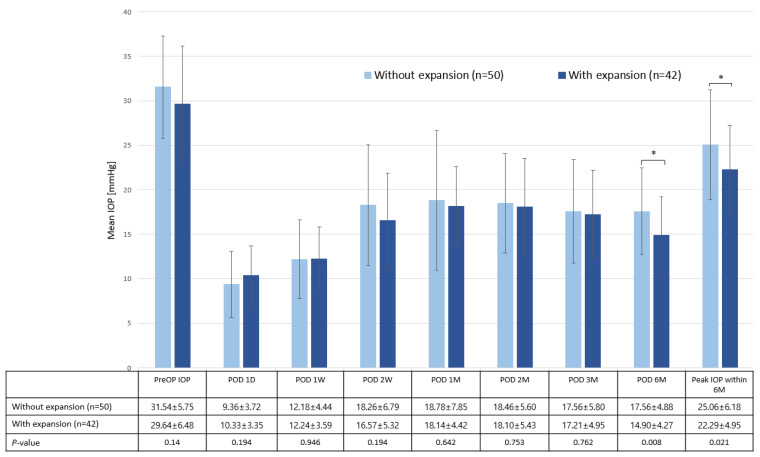
Preoperative and postoperative intraocular pressure (IOP) of the without-expansion and with-expansion (expansion using the pericardium) groups. The bars represent mean ± standard deviation. * *p* < 0.05.

The main outcomes of this study, the 6-month postoperative IOP and peak IOP within the postoperative 6 months, are presented in Figure 3. The preoperative IOP was comparable between the two groups (without-expansion group: 31.54 ± 5.75 mmHg; with-expansion group: 29.64 ± 6.48 mmHg; *p* = 0.14). The 6-month postoperative IOP and peak IOP within the 6 months were lower in the with-expansion group than in the without-expansion group with statistical significance (postoperative 6 months: 17.56 ± 4.88 mmHg vs. 14.90 ± 4.27 mmHg; *p* = 0.008; peak IOP within 6 months: 25.06 ± 6.18 mmHg vs. 22.29 ± 4.95 mmHg; *p* = 0.021). No significant difference was observed in the postoperative complications between the two groups (Table 2). The number of IOP-lowering eyedrops used during the postoperative 6 months were comparable between the two groups, without any statistically significant difference. Bleb width measured at 3 months postoperation was significantly higher in the with-expansion group (4.65 ± 0.78 mmHg) than in the without-expansion group (4.3 ± 0.65 mmHg; *p* = 0.03) (Figure 2B).

The prevalence of a mild hypertensive phase, which was defined as an IOP of ˃ 21 mmHg within the postoperative 3 months, was similar between the two groups (58.0% and 52.4% in the without-expansion and with-expansion groups, respectively; *p* = 0.589). The incidence of both moderate and severe hypertensive phases (defined as an IOP over 25 mmHg and 30 mmHg, respectively, within the postoperative 3 months) was lower in the with-expansion group (16.7% and 4.8%, respectively) than in the without-expansion group (40.0% and 20.0%, respectively; *p* = 0.014 and *p* = 0.031, respectively) (Table 3). 

**Figure 4:**
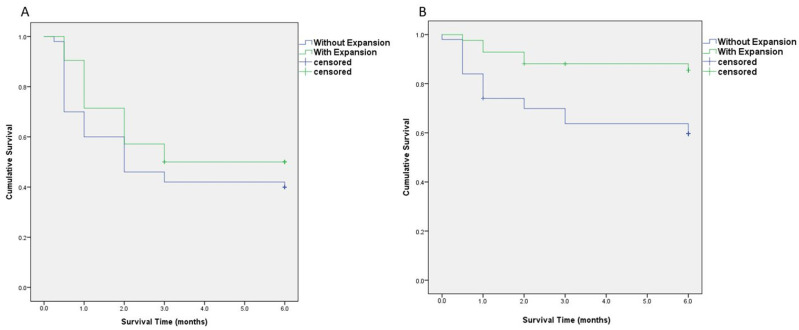
The Kaplan–Meier analysis of the cumulative probability of survival after 6 months post-surgery. (**A**) Survival analysis of qualified success, defined as intraocular pressure (IOP) ˂ 21 mmHg with IOP-lowering eyedrops. The cumulative probability of survival of ˃6 months was 40.0% for the without-expansion group and 50.0% for the with-expansion group (*p* = 0.212). (**B**) For an event defined as IOP of ˃25 mmHg with IOP-lowering eyedrops, the cumulative probability of survival was 60.0% for the without-expansion group and 85.7% for the with-expansion group (*p* = 0.006).

All 50 patients in the without-expansion group completed the follow-up at 6 months postoperation. In the with-expansion group, 3 patients were lost to follow-up; thus, 39 patients completed the follow-up at 6 months postoperation. The qualified success rate at 6 months postoperation was higher in the with-expansion group (89.7%) than in the without-expansion group (74.0%); however, no statistical significance was observed (Table 4). The cumulative probability of survival over the postoperative 6-month period was analyzed using the Kaplan–Meier method (Figure 4). No significant difference was observed for the event “IOP ˃ 21 mmHg with IOP-lowering eyedrops” (*p* = 0.212, Figure 4A); however, for the event “IOP ˃ 25 mmHg,” the cumulative probability of survival was higher in the with-expansion group (85.7%) than in the without-expansion group (60.0%; *p* = 0.006; Figure 4B). 

Logistic regression analyses were performed to determine the factors associated with the occurrence of a moderate hypertensive phase (Table 5). The use of the additional pericardium graft for the expansion of the endplate was the only significant factor associated with the occurrence of a moderate hypertensive phase in univariate (odds ratio 0.300; 95% confidence interval (CI): 0.112–0.807; *p* = 0.017) and multivariate (odds ratio 0.342; 95% CI: 0.124–0.942; *p* = 0.038) analyses.

**Figure 5:**
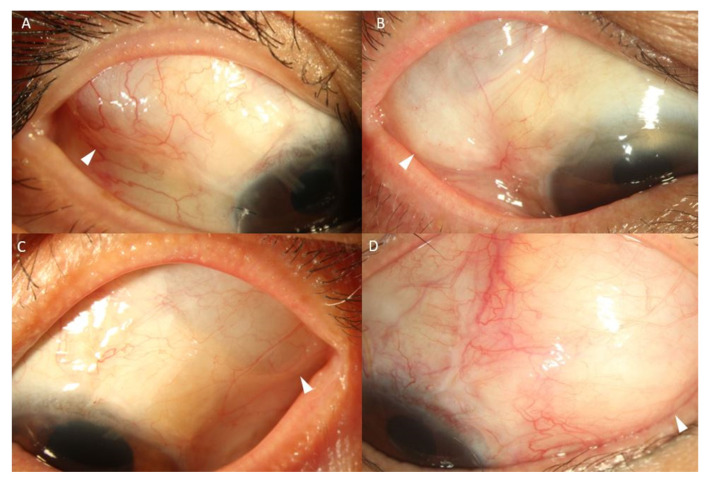
Representative images of patients who underwent the Ahmed glaucoma valve implantation at the superotemporal quadrant at 6 months postoperation; the temporal margins of the blebs are indicated with white arrows. (**A**) Right eye without expansion; (**B**) right eye with the expansion of the endplate surface area using pericardium; (**C**) left eye without expansion; (**D**) left eye with the expansion of the endplate surface area using pericardium.

Representative images of patients at 6 months postoperation are presented in Figure 5. Notably, the with-expansion group (Figure 5B,D) exhibited a relatively wider bleb with more surface area than that of the without-expansion group (Figure 5A,C).

## 4. Discussion

The present study analyzed the short-term surgical outcome of the AGV drainage device implantation when an additional pericardium graft was inserted adjacent to the endplate. The additional pericardium graft was inserted as a space-occupying material, to enlarge the surface area of encapsulation around the endplate, which is a factor associated with the degree of IOP reduction [8,10]. The expanded area resulting from the insertion of the additional graft was indirectly measured based on the bleb width and compared between the two study groups. The additional pericardium was inserted at least 8 mm from the limbus, adjacent to the endplate. Therefore, the bleb width measured at the 4 mm point perpendicular from the limbus is not a direct spreading of conjunctiva by the additional graft itself but the indirect enlargement of surface area as an effect of the additional graft. Bleb width was significantly wider in the with-expansion group than in the without-expansion group (*p* = 0.03). The prevalence of moderate and severe hypertensive phases was significantly less in the with-expansion group than in the without-expansion group (*p* = 0.014 and *p* = 0.031, respectively), while the occurrence of early postoperative complications, such as hypotony, choroidal detachment, and shallow anterior chamber, showed no significant differences between the groups (*p* = 0.411). 

The IOP reduction following GDD implant surgery depends on the bleb wall thickness and total surface area of encapsulation [8]. Our previous study [9] revealed that the maximum bleb wall thickness was thinner in successful AGV implantations than in unsuccessful AGV implantations. Bleb wall thickness is a complex characteristic to modify, determined by a unique foreign body reaction. However, surface area can be relatively easily modified by surgical intervention using either GDDs with larger surface areas or additional surface-area-occupying materials, such as pericardium. Hwang and Kee [13] achieved surface area expansion of AGV implantation by suturing a 300 mm^2^ (30 mm × 10 mm) rectangular pericardial membrane over the AGV and placing its lateral edges under the superior and lateral rectus muscles; consequently, they obtained a lower rate of hypertensive phase without postoperative complications. In this study, we proposed an effective way of expanding the surface area with a less invasive and less time-consuming surgical procedure that does not involve the rectus muscles. We placed the pericardium graft right next to the endplate at the at the sub-Tenon’s space, not involving the rectus muscles when placing it. We also intended to expand the surface area without disrupting the outflow of the aqueous humor by inserting an additional pericardium adjacent to the AVG body rather than directly covering it, thereby expanding the surface area of encapsulation through which aqueous humor passes via passive diffusion to the periocular capillaries and lymphatics [8]. 

The AGV implant has an unidirectional valve mechanism designed to decrease the risk of postoperative hypotony [14], but hypotony occurs despite the presence of the valve mechanism. Ciliary body shutdown resulting in reduced aqueous production, leakage from the area surrounding the tube, and dysfunctional valves were the possible explanations of hypotony with the AGV, the valved GDD [15,16,17]. Tube ligation in AGV implantation is an effective way to reduce the rate of complications related to low postoperative IOP, including hypotony, shallow anterior chamber, and choroidal effusion [17]. Partial ligation of the tube has a similar effect, lowering the rate of hypotony and complications with a similar surgical success compared to without ligation [18]. We partially ligated the tube with releasable sutures in both groups and removed them at 1 week postoperation when there was no sign of hypotony and hypotony-related complications. 

In 1998, Ritch et al. reported using pericardial grafts to cover the subconjunctival tube portion of GDDs [19] to reduce the exposure. The pericardium graft is an allograft frequently used as a surgical material in the ophthalmologic field, especially in glaucoma implant surgeries. The pericardium patch used in this study is commercially available in a 20 mm × 15 mm size. After using 8 mm × 4 mm of the graft as a tube-covering portion, we were able to use the remaining piece for the additional graft (8 mm × 6 mm graft). Although in this study we inserted an 8 mm × 6 mm pericardium graft, the graft size can be customized for each patient according to the periocular space. This study suggests a cost-effective way of reducing the moderate and severe hypertensive phases using a pre-existing, commercially available material. 

The additional pericardium graft resulted in a lower incidence of moderate and severe hypertensive phases. The with-expansion group had a higher complete and qualified success rate than the without-expansion group when combined (97.4% vs. 82.0%, *p* = 0.063) at 6 months postoperation, but there was no statistical significance. This could be explained by the result of a relatively low rate of “complete success” and a high rate of “qualified success”. Based on our previous study [12], we initiated early treatment with aqueous suppressants within the first 3 months when IOP was higher than 18 mmHg to decrease the fibrosis in the bleb and improve the short- and long-term surgical outcomes, and it resulted in less complete success and more qualified success. The postoperative 6-month IOP was significantly lower in the with-expansion group (14.90 ± 4.27 mmHg vs. 17.56 ± 4.88 mmHg; *p* = 0.008), and the number of IOP-lowering eyedrops used after the surgery was comparable between the two groups throughout the 6-month postoperative period. 

Our study had limitations. The two groups had a few differences in baseline characteristics; the without-expansion group had more uveitic glaucoma and secondary glaucoma compared to the with-expansion group (uveitic glaucoma: 20.0% vs. 11.9% and secondary glaucoma: 38.0% vs. 19.0%, *p* = 0.099) when fewer patients in the without-expansion group underwent prior glaucoma surgery than those in the with-expansion group (37.1% vs. 57.4%, *p* = 0.041), implying that the with-expansion group had more refractory glaucoma. The difference in glaucoma subtypes might have affected the surgical outcome. Nevertheless, AGV implantation is proven to be an effective surgical choice in uveitic and secondary glaucoma subtypes [20,21] and we observed a beneficial effect of the additional pericardium, even though the with-expansion group consisted of more refractory glaucoma patients. This was the limitation that had come with the retrospective nature of this study, and further study would be required in a randomized control trial manner. 

The postoperative results were reported only for the first 6 months because many of the patients who showed stable postoperative course were advised to see local clinics since Seoul St. Mary’s Hospital is a tertiary referral hospital in Korea. Only 24 of the without-expansion group and 18 of the with-expansion group made the follow-up visit at 1 year. Of them, the with-expansion group showed a lower 1-year postoperative IOP than the without-expansion group, but there was no statistical significance (17.11 ± 3.12 mmHg vs. 17.96 ± 4.07 mmHg; *p* = 0.507). With this result, the fact that such a small number of patients made up the 1-year follow-up and the patients with a higher IOP or postoperative complications were more likely to be followed-up at 1 year should be taken into account.

Our investigations on the additional pericardium graft only focused on postoperative IOP and bleb width. While we intended to further investigate the bleb wall thickness and intra-bleb configurations, the pericardium graft was too deeply inserted to be amenable for analysis by anterior segment optical coherence tomography. 

In summary, we demonstrated an effective method for expanding the endplate surface area using an additional pericardium graft inserted adjacent to an AGV body. Consequently, moderate and severe hypertensive phases were significantly reduced, and lower postoperative 6-month IOP could be achieved.

## Figures and Tables

**Table 1:** Comparison of baseline characteristics between without-expansion and with-expansion groups.

	Without Expansion (*n* = 50)	With Expansion (*n* = 42)	*p*-Value
Age, years	57.64 ± 15.13	58.40 ± 16.13	0.815
Gender, Male:Female	35:15	28:14	0.732
Laterality, Right:Left	26:24	22:20	0.971
Diabetes mellitus, *n* (%)	9 (18.0%)	5 (11.9%)	0.418
Hypertension, *n* (%)	11 (22.0%)	14 (33.3%)	0.224
Axial length (mm)	25.03 ± 1.68	24.47 ± 1.53	0.127
Central corneal thickness (μm)	549.62 ± 39.86	538.78 ± 47.23	0.241
Lens status, *n* (%)			
Phakic	24 (48.0%)	20 (47.6%)	0.971
Pseudophakic	26 (52.0%)	22 (52.4%)	
Aphakic	0 (0.0%)	0 (0.0%)	
Diagnosis, *n* (%)			
POAG	17 (34.0%)	22 (52.4%)	0.099
PACG/PAC	0 (0.0%)	1 (2.4%)	
Uveitic glaucoma	10 (20.0%)	5 (11.9%)	
PXG	2 (4.0%)	6 (14.3%)	
NVG	2 (4.0%)	0 (0.0%)	
Secondary Glaucoma	19 (38.0%)	8 (19.0%)	
Prior glaucoma surgery, *n* (%)	16 (32.0%)	24 (57.1%)	**0.04**
PreOP glaucoma medication (*n*)	3.88 ± 0.33	3.79 ± 0.42	0.227

NVG = neovascular glaucoma; PAC = primary angle closure; PACG = primary angle-closure glaucoma; POAG = primary open-angle glaucoma; PXG = pseudoexfoliative glaucoma; PreOP = preoperative. Student’s *t*-test and chi-square test were used. Data are mean ± standard deviation unless otherwise indicated. Statistically significant differences between two groups (*p* < 0.05) are indicated in bold.

**Table 2:** Postoperative complications in the without-expansion and with-expansion groups.

		Without Expansion (*n* = 50)	With Expansion (*n* = 42)	*p*-Value
Complications *n* (%)	No complication	38 (76.0%)	28(66.7%)	0.411
	Choroidal detachment	10 (20.0%)	12(28.6%)	
	Shallow anterior Chamber	1 (2.0%)	0 (0%)	
	Hypotony	1 (2.0%)	1(2.4%)	
	Hyphema	0 (0%)	1(2.4%)	
	Exposed tube	0 (0%)	0 (0%)	
	Diplopia	0 (0%)	0 (0%)	

Chi-square test used.

**Table 3:** Hypertensive phase prevalence in the without-expansion and with-expansion groups. Hypertensive phase was classified as mild (>21 mmHg), moderate (>25 mmHg), and severe (>30 mmHg).

Hypertensive Phase	Without Expansion (*n* = 50)	With Expansion (*n* = 42)	*p*-Value
Mild (>21 mmHg), *n* (%)	29 (58.0%)	22 (52.4%)	0.589
Moderate (>25 mmHg), *n* (%)	20 (40.0%)	7 (16.7%)	**0.014**
Severe (>30 mmHg), *n* (%)	10 (20.0%)	2 (4.8%)	**0.031**

Student’s *t*-test is used. Statistically significant differences between two groups (*p* < 0.05) are indicated in bold.

**Table 4:** Comparison of success rate (%) between without-expansion and with-expansion group at 6 months postoperation. Complete success was defined as sufficient intraocular pressure (IOP) lowering (<21 mmHg) without any IOP-lowering eyedrops. Qualified success was defined as IOP < 21 mmHg with IOP-lowering eyedrops. Failure was defined as IOP over 21 mmHg even with IOP-lowering eyedrops.

		Without Expansion (*n* = 50)	With Expansion (*n* = 39)	*p*-Value
Success Rate *n* (%)	Complete	4 (8.0%)	3 (7.7%)	0.063
	Qualified	37 (74.0%)	35 (89.7%)	
	Failure	9 (18.0)%	1 (2.5%)	

Chi-square test used.

**Table 5:** Logistic regression analysis of factors associated with hypertensive phase over 25 mmHg.

Variables	Univariate		Multivariate	
	Odds Ratio (95% CI)	*p* Value	Odds Ratio (95% CI)	*p* Value
Age, years	0.979 (0.951–1.007)	0.144		
Diabetes mellitus	0.957 (0.272–3.364)	0.945		
Hypertension	1.189 (0.440–3.213)	0.733		
Axial length	1.099 (0.820–1.472)	0.527		
Central corneal thickness	0.998 (0.987–1.008)	0.672		
Lens status	1.212 (0.492–2.986)	0.676		
Prior glaucoma surgery	0.434 (0.166–1.132)	0.088	0.546 (0.201–1.484)	0.236
PreOP IOP	1.007 (0.936–1.083)	0.857		
PreOP medication no.	1.811 (0.468–7.015)	0.390		
Additional pericardium graft	0.300 (0.112–0.807)	**0.017**	0.342 (0.124–0.942)	**0.038**
Bleb width	0.647 (0.322–1.300)	0.221		

PreOP = preoperative, IOP = intraocular pressure, no. = number. Values with statistical significance are shown in bold.

## Data Availability

Data may be provided upon reasonable request.
